# DNA Loss at the *Ceratocystis fimbriata* Mating Locus Results in Self-Sterility

**DOI:** 10.1371/journal.pone.0092180

**Published:** 2014-03-20

**Authors:** P. Markus Wilken, Emma T. Steenkamp, Michael J. Wingfield, Z. Wilhelm de Beer, Brenda D. Wingfield

**Affiliations:** 1 Department of Genetics, Forestry and Agricultural Biotechnology Institute, University of Pretoria, Pretoria, South Africa; 2 Department of Microbiology and Plant Pathology, Forestry and Agricultural Biotechnology Institute, University of Pretoria, Pretoria, South Africa; Soonchunhyang University, Republic of Korea

## Abstract

Fungi have evolved a remarkable diversity of reproductive strategies. Some of these, most notably those of the model fungi, have been well studied but others are poorly understood. The latter is also true for uni-directional mating type switching, which has been reported in only five fungal genera, including *Ceratocystis*. Mating type switching allows a self-fertile fungal isolate to produce both self-fertile and self-sterile offspring. This study considered the molecular nature of uni-directional mating type switching in the type species of *Ceratocystis*, *C. fimbriata*. To do this, the genome of *C. fimbriata* was first examined for the presence of mating type genes. Three mating genes (*MAT1-1-1*, *MAT1-2-1* and *MAT1-1-2*) were found in an atypical organisation of the mating type locus. To study the effect that uni-directional switching has on this locus, several self-sterile offspring were analysed. Using a combination of next generation and conventional Sanger sequencing, it was shown that a 3581 base pair (bp) region had been completely deleted from the *MAT* locus. This deletion, which includes the entire *MAT1-2-1* gene, results in the permanent loss of self-fertility, rendering these isolates exclusively self-sterile. Our data also suggest that the deletion mechanism is tightly controlled and that it always occurs at the same genomic position. Two 260 bp direct repeats flanking the deleted region are strongly implicated in the process, although the exact mechanism behind the switching remains unclear.

## Introduction

The key role that reproduction plays in the survival of a species has undoubtedly contributed to the remarkably broad range of reproductive strategies encountered across the fungal kingdom [1]–[3]. Several of these are found in model fungal species where they have been intensively studied and used to formulate broad hypotheses regarding mating in the fungi [4]–[10]. Recent technological advances such as next generation sequencing and improved bioinformatic capabilities have provided tools to expand these studies to include the molecular nature of reproductive strategies in more fungi, including non-model species. These studies have led to the discovery of mating strategies that differ significantly from those of model species [11], [12]. Consequently, they have broadened our understanding of sexual reproduction in the fungal kingdom, thereby contributing to knowledge regarding the evolution of sex in eukaryotes in general [13]–[15].

Sexual reproduction in the fungal kingdom can take many different forms [16]. Mating in the sac fungi (phylum Ascomycota) represents a bipolar system [2], [17] under the control of a single mating locus (*MAT-1*) with two allelic or idiomorphic forms (*MAT1-1* and *MAT1-2*) [10]. In heterothallic species, sexual reproduction can proceed only when the interacting individuals encode different but complementary sets of genes at their *MAT-1* locus [8], [17]. The finer details of this heterothallic and bipolar mating system have been the subject of numerous studies in the model fungus, *Neurospora crassa* [5], [18], [19]. Species where a single isolate can complete the sexual cycle independently is referred to as homothallism [2]. In the homothallic fungus *N. pannonica* this is accomplished by the presence of both mating idiomorphs in the same haploid genome [20]. Other species such as *N. tetrasperma* employ a novel mating system where two copies of the haploid genome, each carrying different mating idiomorphs, occur in a single ascospore, which germinates to produce a self-fertile culture. This mating system is referred to as pseudohomothallism [2], [8], [21].

Intriguingly, some fungi are capable of switching their mating types or mating specificities [4], [22]–[26]. Isolates of the budding yeast *Saccharomyces cerevisiae* can grow vegetatively as either a *MATa* or *MATα* haploid cells, or as *MATa*/*MATα* diploid cells [4]. However, only the diploid cells are capable of meiosis and sexual reproduction [27]. Through bi-directional mating type switching a haploid cell of one mating type (either *MATa* or *MATα*) can give rise to haploid progeny of both mating types (i.e. *MATa* and *MATα* cells) [4]. This results in a mixed culture from which diploids can form, allowing sexual reproduction to proceed. The entire bi-directional mating type switching process is finely orchestrated and involves the deletion of DNA at the *MAT* locus and replacement with a copy of the opposite idiomorph [4], [27]. The information that is copied into the locus is provided by a silent version found elsewhere within the yeast genome [4].

Uni-directional mating type switching is an irreversible switching mechanism that results in the production of both self-fertile and self-sterile isolates by a homothallic species [28], [29]. Once uni-directional switching has occurred, the ‘switched’ strain is self-sterile and cannot revert back to being self-fertile and homothallic. Uni-directional mating type switching has not been closely studied, and is known in only four fungal genera, *Ceratocystis* (class Sordariomycetes), *Glomerella* (class Sordariomycetes), *Chromocrea* ( = *Hypocrea*) (class Sordariomycetes), and *Sclerotinia* (class Leotiomycetes) [22], [28], [29]. A mixture of both self-fertile and self-sterile individuals results from the switching event that apparently occurs during sexual reproduction [30]. Although the molecular basis for this process is not well understood, it has recently been suggested that a DNA inversion event such as that studied in *Sclerotinia sclerotiorum* could result in altered gene expression of the *MAT* genes leading to the observed effects such as self-sterility [11]. Studies on a few *Ceratocystis* species have suggested that uni-directional switching might involve the loss or rearrangement of at least one mating gene (*MAT1-2-1*) [28], [29].

The aims of this study were to (i) fully describe the *MAT-1* locus of a *Ceratocystis* species capable of uni-directional mating type switching and (ii) to characterize the genomic region that is affected during the switching process. For this purpose, we used the type species of *Ceratocystis*, *C. fimbriata*, for which whole-genome sequence information is available for a self-fertile isolate (GenBank accession number APWK00000000) [31]. We also sequenced the genome of a self-sterile isolate and utilized sequence comparisons in conjunction with standard DNA-based and mycological methods to elucidate the structure of the *MAT* locus for self-fertile and self-sterile isolates. The results provide a further insight into the molecular basis of uni-directional switching and the DNA affected during this unusual process.

## Results

### Selection of Self-fertile and Self-sterile Isolates

For *Ceratocystis*, single isolates in pure culture that produce sexual structures called ascomata are referred to as being self-fertile [28]. Progeny produced from a single spore taken from these ascomata can either be self-fertile, or alternatively self-sterile. Such self-sterile strains of *Ceratocystis* do not produce ascomata in pure culture [28], [29]. Both of the self-fertile isolates of *C. fimbriata* (CMW14799 and CMW1547*)* used in this study produced abundant ascomata with ascospore drops within 3–4 weeks after first, second and third transfer, indicating the occurrence of sexual reproduction. The sub-culturing of spore drops ensured the retention of self-fertility. Single ascospore isolates prepared from these spore masses either produced cultures with the ability to produce ascomata, or cultures that grew only vegetatively without producing ascomata. Eleven isolates (designated CMW14799_B1–11) originating from CMW14799 and six isolates (CMW1547_B1–6) from CMW1547 that did not produce ascomata were selected for further study. These self-sterile strains could not be induced to form ascomata despite multiple rounds of sub-culturing, even when incubated on media containing thiamine known to promote ascomatal development [32], [33].

### Ceratocystis Fimbriata Genome and MAT Genes

Within the available genome sequence for the self-fertile isolate CMW14799 [31], we identified four contigs possibly containing *MAT-1* genes (Table S1) using tBLASTn searches from the CLC genomics workbench. Several open reading frames (ORFs) were predicted within these contigs using AUGUSTUS [34]. The results of the BLASTp searches against the database of the National Center for Biotechnology Information (NCBI; http://www.ncbi.nlm.nih.gov) predicted that the single ORF in contig 2280 could encode a protein substantially similar to the *MAT1-2-1* HMG (High Mobility Group) box fragment, previously obtained from *C. fimbriata* [29]. The first ORF in contig 02573 was predicted to encode a product with a high level of similarity to the MAT1-1-2 protein, while the second ORF was predicted to encode a protein similar to a conserved hypothetical protein predicted in *Verticillium alboatrum*. The predicted product of the third ORF on contig 02573 showed similarity to an Importin-beta domain protein in *Glomerella graminicola*, although it was only a partial prediction, lacking an in-frame stop codon. Six ORFs were predicted from contig 02036, of which the first showed no similarity to any gene in the NCBI database and was designated as a hypothetical protein. The products predicted by the following four ORFs showed similarity to Cytochrome c oxidase sub-unit VIa, APN2 DNA lyase, the APC anaphase promoting complex gene, and the SLA2 cytoskeleton assembly protein, respectively. The predicted *MAT1-1-1* gene was positioned after these ORFs. The *de novo* AUGUSTUS predictions suggested that contig 02181 contained seven ORFs. While the initial tBLASTn analysis indicated similarity to a MAT1-1-3 protein, on closer examination, none of the predicted products on contig 02181 showed any significant similarity to this or any other MAT-1 protein. Reference assembly of sequence data from isolate CMW14799_B11 to contigs 02036, 02573 and 02280 of isolate CMW14799 allowed identification of putative *MAT1-1-1*, *MAT1-1-2*, *SLA2*, *APC* and *APN2* genes in the self-sterile isolate.

The conserved alpha-box (for MAT1-1-1), HMG-box (for MAT1-2-1) and PPF/HPG (for MAT1-1-2) domains inferred for the respective *C. fimbriata* ORFs were very similar to those of known fungal *MAT* genes (Figure S1). Furthermore, both the *MAT1-1-1* and *MAT1-2-1* genes contained an intron at conserved positions described previously [12], [35], [36]. However, the predicted *C. fimbriata* MAT1-1-2 protein had a PYF domain rather than the usual PPF (proline-proline-phenylalanine) domain [12]. The TAC codon responsible for this change had a high coverage in the CMW14799_B11 reference assembly (50x) and when subjected to Sanger sequencing (10x). Despite this difference, a BLASTp analysis of the predicted protein showed similarity (E = 4.62E-6) to other fungal *MAT1-1-2* proteins, and apart from the proline-tyrosine change, several other amino acids in the conserved region of the protein showed identity to known MAT1-1-2 domains (Figure S1).

### Reconstruction of the *Ceratocystis Fimbriata MAT-1* Locus

Primers designed from the predicted *MAT1-1-1*, *MAT1-1-2* and *MAT1-2-1* gene sequences were used to amplify fragments from all isolates used in this study (Table S2). Other than for *MAT1-2-1*, amplicons were produced for every gene region and from all isolates. The *MAT1-2-1* fragment was amplified only from the self-fertile isolate CMW14799, while the other fragments were produced regardless of the sexual state of the isolate.

**Figure 1:**
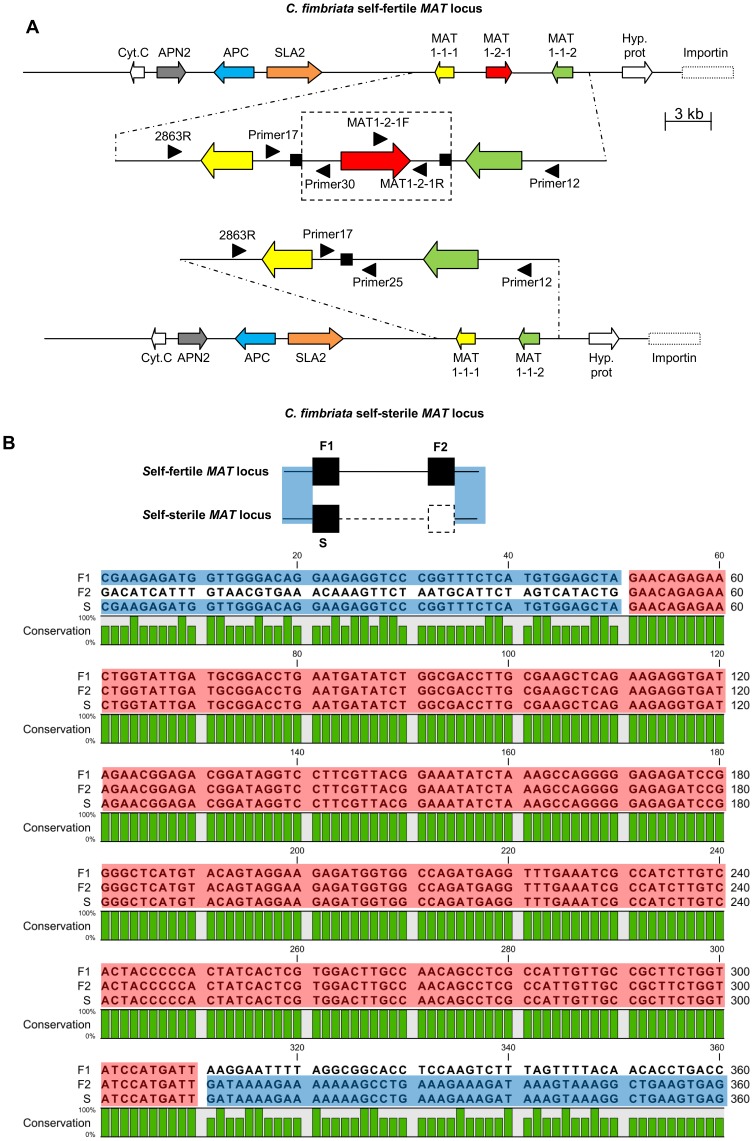
The mating locus (*MAT-1*) and 260 bp repeat motif sequence of self-fertile and self-sterile isolates of *Ceratocystis fimbriata*. A. The *MAT-1* locus and flanking regions of a *C. fimbriata* self-fertile (top) and self-sterile (bottom) isolate, drawn to the indicated scale. During the switching event 3581 bp (including the entire *MAT1-2-1* gene and 1911 bp of flanking sequence) is lost. Genes are depicted by block arrows, with the arrow head indicating the direction of the ORF. Enlarged sections (not drawn to scale) show the *MAT* genes. Black blocks indicate the position of the direct repeat regions, while the dashed block indicates the region lost during a switching event. Black arrow heads indicate the position and direction of primers used for analysis of the *MAT* locus. B. A simplified version of the self-fertile and self-sterile *MAT* locus of *C. fimbriata*. Solid blocks and lines indicate repeats and genomic sequence, respectively. F1 and F2 refer to the first and second copy of the repeat in the self-fertile locus, and S to the single copy of the repeat in the self-sterile locus. Dashed blocks and lines indicate the region deleted in the self-sterile isolates. Shading corresponds to that of the alignments. Bottom - An alignment of both versions of the 260 bp repeat from a self-fertile isolate to the same repeat region in self-sterile isolates. Included in the alignment is 50 bp of sequence linked to both sides of all the repeats for sequence comparison. The section of the self-fertile locus to the left of F1 is 100% identical to the region left of repeat S in the self-sterile isolate. Similarly the region following to the right of repeat S in the self-sterile isolate is 100% identical to the sequence right of repeat F2 in the self-fertile isolate. The bottom green bar graph indicates the amount of similarity between the sequences. This indicates the repeats F1, F2 and S are identical in sequence (100% similarity). Blue blocks show the areas of sequence identity between regions outside of the repeats. Red blocks indicate the repeats.

A long-range PCR for self-sterile isolate CMW14799_B11 using primer set 2863R/Primer12 produced a single PCR product (size 8448 bp) that spanned the *MAT* locus and left and right flanking regions (Figure 1A). However, no product was produced using the primer sets 2863R/MAT1-2-1R and MAT1-2-1F/Primer12 (Figure 1A). For the self-fertile isolate CMW14799, fragments were produced for all three primer sets. For these PCRs, single bands were produced with the 2863R/MAT1-2-1R (size 6531 bp) and MAT1-2-1F/Primer12 (size 5992 bp) primer sets, while primer set 2863R/Primer12 amplified two fragments. The larger of these fragments (size 12029 bp) corresponded to the predicted size of the self-fertile *MAT-1* locus, which contained all three *MAT-1* genes. The smaller fragment was the same size as what was observed for the self-sterile locus in isolate CMW14799_B11. The data thus suggest that CMW14799 contains a mixture of the self-fertile and self-sterile *MAT-1* locus, because the DNA of this isolate was extracted from a culture producing ascomata and ascospores.

All the sequence information for self-fertile isolate CMW14799 produced a region that spanned 46416 base pairs (bp) (GenBank accession number KF033902) and contained all of the *MAT-1* genes investigated in this study. Reconstruction of the corresponding region in the self-sterile isolate (CMW14799_B11) generated a 42835 bp sequence (GenBank accession number KF033903) that was significantly shorter than that in the self-fertile isolate. The 46416 bp region in the self-fertile isolate included three genes in the order *MAT1-1-1*/*MAT1-2-1*/*MAT1-1-2*. The genes encoding SLA2, APC, DNA Lyase and Cytochrome c oxidase subunit VIa were linked to the left of the *MAT* genes, while the genes encoding a conserved hypothetical protein and part of an Importin-beta domain-containing protein were found linked to the right of the mating genes (Figure 1A). Comparisons to the 42835 bp region in the self-sterile isolate showed identical gene order and nucleotide sequence to the self-fertile isolate apart from a fragment of 3581 bp (consisting of the *MAT1-2-1* gene and flanking regions) that was missing. Apart from a 260 bp repeat region (see below), BLAST searches of the *de novo* assembled CMW14799_B11 genome could not reliably (E≤0.00) match any region in the extra 3581 bp fragment present in the self-fertile genome. The *MAT-1* locus was defined as the region that extended from the start of the *MAT1-1-1* gene to the end of the *MAT1-1-2* gene. In the self-fertile isolate, this corresponded to a 8928 bp region that included three genes (*MAT1-1-1*, *MAT1-2-1* and *MAT1-1-2*), and in the self-sterile isolate, it corresponded to a 5014 bp region that consisted of two genes (*MAT1-1-1* and *MAT1-1-2*) (Figure 1A).

Sequence analysis of the *MAT-1* locus showed that the 3581 bp region deleted during mating type switching (Figure 1A) included the full *MAT1-2-1* gene and 1911 bp of non-coding sequence flanking this gene. RepFind analysis [37] identified the presence of several repeat units with a p-value of 0.00 (Table S3). One of these was a direct repeat that appeared twice and consisted of 260 nucleotides, which flanked the boundaries of the deleted region in CMW14799 at positions 28768 and 32349. BLAST analysis of the original *C. fimbriata* CMW14799 genome sequence [31] identified only a single 261 bp contig (contig 02281) that contained the full 260 bp repeat, although it was not identified in any of the contigs containing *MAT* information (i.e., contigs 02036, 02573 and 02280). The 260 bp sequence was also present in a single copy in the reconstructed *MAT-1* locus of the self-sterile isolate CMW14799_B11, between the *MAT1-1-1* and *MAT1-1-2* genes at position 28768. This repeat had 100% sequence identity to that seen in the self-fertile isolates (Figure 1B). Analysis of the full self-fertile region (46416 bp) using the program REPuter [38] also identified the same 260 bp repeat (Table S3). This repeat showed no similarity to any known repeat sequence present in the NCBI nucleotide database or the RepBase repeat database.

### A Specific Stretch of DNA is always Lost during Mating Type Switching

PCR analysis on an additional self-fertile and five additional self-sterile isolates yielded the expected fragments as described above. All fragments were cloned and sequenced using Sanger technology. These sequences of self-fertile isolate CMW1547 was assembled into an 11990 bp fragment that showed 100% sequence identity with the corresponding sequence from self-fertile isolate CMW14799. This region included the 8928 bp *MAT-1* locus and two copies of the 260 bp direct repeat. The corresponding region in all of the self-sterile isolates was 8409 bp in size and was again identical to that found for isolate CMW14799_B11. Sequence comparisons showed that all self-sterile fragments were 100% identical in sequence, regardless of the parent isolate (CMW14799 vs. CMW1547), and contained a single copy of the 260 bp repeat region in the 5014 bp *MAT-1* locus.

The PCR primer sets Primer17/Primer30 and Primer17/Primer25 proved helpful in evaluating the structure of the *MAT-1* locus of the *C. fimbriata* isolates (Figure 1A). PCR with Primer17 and Primer30 produced a band of 591 bp only in the self-fertile isolates, while Primer17 and Primer25 produced an 855 bp product in the self-sterile isolates. PCR analyses of the two self-fertile isolates and six self-sterile isolates described above, as well as an additional 11 self-sterile isolates produced the expected size bands. Sequencing of these amplicons for selected isolates confirmed that the products corresponded to the relevant *MAT-1* regions. These primer pairs can therefore be used to confidently assign isolates as either self-fertile (presence of product Primer17/Primer30) or self-sterile (presence of product Primer17/Primer25).

## Discussion

The existence of self-fertile and self-sterile isolates in *C. fimbriata* has been known for more than 60 years [30], [39]. The process that allows self-fertile isolates to give rise to both self-fertile and self-sterile forms after sexual reproduction is known as uni-directional mating type switching [22]. Previous studies have suggested that at least part of the *MAT1-2-1* gene is lost during the switching event in *Ceratocystis* species [28], [29]. However, compared to other common modes of sexual reproduction in fungi such as heterothallism and homothallism [2], [8], [15], uni-directional mating type switching has been minimally studied and its molecular basis is poorly understood. In this study, the *MAT-1* locus of two self-fertile and several self-sterile isolates of *C. fimbriata* was fully characterised. Subsequent analysis showed that during the switch from self-fertility to self-sterility, a large part of the *MAT-1* locus that includes the *MAT1-2-1* gene is completely lost from the genome. Apart from the region that is deleted, the remainder of the locus and regions flanking it is identical in gene order and sequence between self-fertile and self-sterile isolates.

The gene organisation of the *MAT-1* locus and its flanking regions in *C. fimbriata* is unusual. Although the *MAT1-1-1* and *MAT1-2-1* genes were present in the self-fertile isolates as expected [2], [8], no *MAT1-1-3* gene was identified. Although the latter gene is often present in other members of the class Sordariomycetes [12], [40], some examples of this gene being absent in homothallic [41] and heterothallic [42], [43] species exist. The *C*. *fimbriata MAT1-2-1* gene was flanked by *MAT1-1-1* and a second *MAT1-1* gene, *MAT1-1-2* (Figure 1A), which is a peculiar arrangement for homothallic fungi. In addition, the SLA2 and APN2 genes usually flank the *MAT-1* locus [44]–[47], but in *C. fimbriata* both were located upstream of *MAT1-1-1* where they flanked the APC gene. While the APC gene has been associated with the *MAT* region in other fungi [36], [48], [49], to the best of our knowledge, it has not previously been reported to be positioned between the genes encoding APN2 and SLA2. Examples where the SLA2 and APN2 genes are found linked to the side of the *MAT* locus is known, but in these species the genes flank a *COX13* gene instead of the APC gene [50]. In *Mycosphaerella fijiensis*, and *My. graminicola* (pathogens of banana and wheat, respectively) the APC gene is found upstream of the DNA lyase gene [36], [48]. The *MAT* locus of *C. fimbriata* is thus similar to those previously reported for Ascomycota, although some significant gene rearrangements have occurred in the position of the individual genes.

The mating genes in *C. fimbriata* showed similarity in amino acid sequence and conserved intron positions when compared to other known fungal *MAT* proteins. The alpha-box domain in *MAT1-1-1* contained an intron in a conserved position considered to be characteristic of this mating gene [12], [36]. Similarly, a conserved intron position across a serine residue in the *MAT1-2-1* gene HMG-box [12], [35] provided confidence in the identity of this protein. Although no characteristic conserved domain or intron position has been described for the *MAT1-1-2* gene, some studies have suggested the presence of a PPF [12] or HPG [51] motif as characteristic of the protein. *Ceratocystis fimbriata* deviates from both these motifs because the common middle proline in both are replaced by a tyrosine, making the motif PYF (vs. PPF) and HYG (vs. HPG). This is the first MAT1-1-2 protein described from the order Microascales and it is possible that this difference could be characteristic of this group of fungi.

In this study, we have shown that a self-fertile isolate of *C. fimbriata* is able to switch to a self-sterile phenotype by losing the *MAT1-2-1* gene possibly through that involvement of a 260 bp direct repeat. This gene is not only missing from the *MAT-1* locus, but is entirely deleted from the genome in self-sterile isolates. Co-segregation of self-sterility and loss of the *MAT1-2-1* gene has been suggested previously [29] and hypotheses to explain this observation include complete loss of the *MAT1-2-1* gene [28], [29], transposition and subsequent expression of gene information from silent *MAT* loci [22] or genome rearrangements by mechanisms such as inversion [11]. Although PCR and Southern Blot analysis targeting a fragment of *MAT1-2-1* previously showed that at least part of the coding sequence was lost [29], the present study is the first to provide sequence evidence for the complete deletion of the entire gene and flanking regions from the *MAT* locus. Our findings further suggest that this deletion is in some way associated with a repeated motif of 260 bp that flanks the *MAT1-2-1* gene in self-fertile individuals, and a single copy of this motif is present in self-sterile individuals. This repeat motif does not show any similarity to other described repeat motifs or known transposable elements, and seems to be a novel repeat element unique to the *C*. *fimbriata MAT* locus. The assembled *C. fimbriata* genome available in the NCBI database contained only a single contig with the 260 bp repeat. This contig was unrelated to those containing the *MAT* gene information, and is most likely due to the well-described inability of *de novo* genome assembly algorithms to deal with repeat regions [52]. The use of Sanger sequencing with longer read lengths allowed us to resolve this issue in both the self-fertile and self-sterile *MAT* locus. In comparison to the self-fertile isolates, the 260 bp repeat motif is present in only a single copy in the *MAT* region of the CMW14799_B11 genome.

A recombination event around the 260 bp repeats could result in deletion of the *MAT1-2-1* gene, leaving a single copy of the repeat sequence. A possible mechanism to explain this is a pre-meiotic recombination event, anchored by the 260 bp direct repeat sequences present in the self-fertile *C. fimbriata MAT* locus. This would yield a product containing two copies of the *MAT1-2-1* gene with a copy of the repeat sequence between these. This sequence would be unstable during meiosis, and the resultant meiotic products would contain either a single copy of the gene flanked by the repeat region (the self-fertile locus) or a *MAT* locus with a single copy of the repeat lacking the *MAT1-2-1* gene (the self-sterile locus). The involvement of pre-meiotic recombination is also in agreement with previous suggestions that the switching event might occur in the protoperithecium of *C. coerulescens* [28]. From a mechanistic point of view, our model for the process in *C. fimbriata* is similar to that described for *Podospora anserina* and *N. crassa* [53], [54]. Detailed analysis of transformants of these fungi revealed that artificially introduced duplicates of specific target sequences were lost during meiosis, despite being maintained during vegetative growth. Pre-meiotic recombination events anchored by homologous sequences are the basis of this loss, and produces products similar to those seen during mating-type switching in *C. fimbriata*.

The loss of DNA during uni-directional mating switching in *C. fimbriata* is especially intriguing because few examples of controlled DNA deletion from the genomes of Eukaryotes are known. In *C. fimbriata,* every mating cycle is an independent event and the DNA loss is controlled during every round of switching. Although this process might at first seem comparable to that in *S. cerevisiae* [4], major differences between these fungi exist. The latter species has two silent copies of the *MAT-1* information in addition to the functional *MAT-1* locus. It is thought that the HO restriction enzyme (expressed only during the G1 phase in mother cells [2]) targets a 24 bp region to produce a double-stranded break, thus initiating recombination and copying of the *MAT* information from one of the silent copies into the *MAT-1* locus [4], [55], [56]. There are two important differences between this process and the one that we have illustrated for *C. fimbriata*. Firstly, the *C. fimbriata* genome does not contain detectable silent copies of the *MAT-1* locus or any of the genes for which it encodes. Secondly, the *C. fimbriata* switch is irreversible and the lost *MAT* information is never replaced. The divergent nature of the processes is further supported by the fact that the HO gene or pseudogene has been reported only in members of the Saccharomycotina [2]. In addition, tBLASTn searches of the *C. fimbriata* genome failed to identify any nucleotide sequences that could possibly encode an enzyme homologous to the *S. cerevisiae* HO enzyme (data not shown). The process of uni-directional switching in *C. fimbriata* is thus clearly distinct from, and not comparable with bi-directional switching seen in *S. cerevisiae*. In this regard, uni-directional switching might not be an appropriate description of the process because the deletion results in a change from self-fertility to self-sterility, rather than a change of mating type (e.g. from *MAT1-1* to *MAT1-2*).

A preliminary analysis of the *MAT* region of *Chromocrea spinulosa* ( = *Hypocrea spinulosa*), another ascomycete known to have uni-directional mating type switching [22], [26], revealed the presence of two repeats flanking the region lost during switching [57]. It has been reported that the *Ch. spinulosa MAT-1* region contains three *MAT1-1* genes in the order *MAT1-1-1*, *MAT1-1-2*, *MAT1-1-3* in both self-fertile and self-sterile strains. Linked to this region, a *MAT1-2-1* gene is present only in self-fertile strains, flanked by two copies of the repeat. The same repeat is present in a single copy in the self-sterile *MAT* locus, indicating that both the *MAT1-2-1* gene and the second copy of the repeat were deleted from the genome. The repeat sequence reported cannot be compared to the 260 bp repeat from the *C. fimbriata MAT* locus because the *Ch. spinulosa* mating sequences are not publicly available. The striking similarity in the structure of the *MAT-1* locus in *C. fimbriata* and *Ch. spinulosa* might suggest that the model for DNA loss that we have proposed for uni-directional switching is conserved more widely in the Ascomycota than might presently be assumed.

The role of uni-directional mating switching in the life-cycle of *C. fimbriata* and its relatives that share this unusual reproductive strategy remains unknown. Varying ratios of self-fertile to self-sterile progeny from a single ascoma (ranging from 1∶1 to 9∶1) has been reported for species of *Ceratocystis* [28], [30]. Previous studies [58] have also shown that the self-sterile progeny grow more slowly than those having the self-fertile *MAT* type, and this was also observed in the present study (results not shown). It has been suggested that this apparent decrease in fitness could be attributed to more genes being lost during the switch [28], [58]. Complete genome comparisons are needed to determine whether this is indeed the case. Another explanation for the decreased fitness of self-sterile isolates could be the consequence of pleiotropic effects associated with the loss of the *MAT1-2-1* gene [28], [29], [58]. This is because the *MAT1-2-1* gene encodes a transcription factor, presumably involved in the regulation and expression of a number of downstream genes, conferring sexual identity to MAT1-2 isolates [2], [8], [59]. An additional hypothesis would be that the production of self-sterile isolates during uni-directional switching could promote preferential outcrossing of this fungus. The ability of self-sterile cultures to mate with self-fertile isolates has been observed previously in the related species *C. coerulescens* [28]. Outcrossing brought about in this way would allow the fungus a mechanism to avoid the progressive accumulation of deleterious mutations associated with selfing or clonal reproduction [20]. Moreover, some *Ceratocystis* species produce a second type of self-sterile isolate that harbours an apparently functional *MAT1-2-1* gene in its *MAT-1* locus [28], [30]. These self-sterile isolates could provide additional compatible mating partners for the self-sterile isolates lacking a *MAT1-2-1* gene. If both types occur in nature, this would promote outcrossing, allowing *Ceratocystis* to fully exploit the benefits of sexual reproduction.

## Materials and Methods

### Fungal Isolates

Two isolates (CMW14799 and CMW1547) of *C. fimbriata* were used in this study (Table S4). To induce sexual reproduction and the consequent formation of sexual structures called ascomata, isolates were grown on 2% malt extract agar (MEA-TS; 20 g L^−1^ malt extract (Biolab, Merck), 20 g L^−1^ agar (Biolab, Merck)) supplemented with thiamine (100 mg/l) and streptomycin (150 mg/l) at room temperature for 2–4 weeks. The successful production of ascomata indicated that both cultures were self-fertile. These cultures were maintained for three generations on MEA-TS by sub-culturing ascospore masses that were produced on the apices of mature ascomata. To produce single ascospore isolates, a sterile needle was used to collect ascospore masses from first, second and third round sub-cultures. These masses were dissociated in 50 μl isoparaffin solvent, Soltrol 130 (Chemfit, Gauteng, South Africa), and streaked onto the surface of 1% MEA in Petri dishes. After overnight incubation at room temperature, single hyphal tips were cut from germinating ascospores and transferred onto 2% MEA-TS. This process was repeated three times and when no ascomata was formed after 4 weeks of incubation (indicating the absence of sexual reproduction), the isolates were considered as self-sterile, and assigned a _B post-script (e.g. CMW14799_B1). In this way 17 single ascospore self-sterile isolates (CMW14799_B1–B11 and CMW1547_B1–B6) were generated and used here (Table S4).

### *Ceratocystis Fimbriata* Genome and *MAT* Genes

Local BLAST searches were used to identify contigs containing *MAT-1* genes in the genome sequence of *C. fimbriata* isolate CMW14799 (GenBank accession number APWK00000000) by making use of the CLC genomics workbench version 4.8 (CLC bio, Denmark). Known protein sequences for MAT1-1-1, MAT1-1-2, MAT1-1-3 and MAT1-2-1 (Table S1) were used as query sequences in a tBLASTn search against all contigs of the *C. fimbriata* genome. All *MAT-1* gene-containing contigs were then subjected to *de novo* protein predictions using the AUGUSTUS online interface [34] and the *Fusarium graminearum* gene models. All the predicted protein sequences were compared using BLASTp to those in NCBI protein database for putative identification (Table S1). The conserved domains of the MAT1-1-1, MAT1-1-2 and MAT1-2-1 proteins were defined based on the work done by Kanematsu and colleagues [12] and compared to that of other known fungal *MAT* genes (Figure S1). To do this the conserved regions were aligned using MAFFT [60] and inspected for similarities in the CLC Main Workbench.

The genome of the self-sterile isolate CMW14799_B11 was sequenced to allow for comparison of the *MAT-1* locus between a self-sterile and self-fertile isolate. The DNA used for this was extracted using the method described by Barnes *et al*. [61] and single reads of 100 bp each were produced using the Illumina HiScanSQ Platform (Biotechnology Platform, Agricultural Research Council, South Africa). The quality of these reads was assessed using the “Create sequencing QC report” command of the CLC Genomics workbench. Sequencing produced *ca.* 3.4 Gb of sequence located on *ca.* 35 million reads. These reads were of high quality with 76% of the reads having a PHRED score of ≥30, and this number increased to 88% for a PHRED score of ≥25. To assemble and identify the *MAT-1* genes in this individual, raw sequence reads were reference-mapped to the *MAT-1* gene-containing contigs of CMW14799 (Table S1) using the CLC Genomics Workbench software package. The full complement of raw reads and the following settings were used for the mapping: Mismatch cost set to 2, Insertion cost set to 2, deletion cost set to 3, length fraction set to 0.5, similarity fraction set to 0.8 and non-specific matches were handled by random mapping. Contig 02036 had 99× coverage while the 02573 contig showed 85× coverage. However none of the sequence reads mapped or assembled to the 02280 contig. The *MAT-1* locus and flanking genes were defined by similarity to the self-fertile locus and through BLASTp searches of the NCBI database as described above. In addition, all the reads produced during sequencing was trimmed for quality using CLC genomics with the quality threshold set to 0.05 and the option to trim ambiguous nucleotides selected (maximum number of ambiguous bases allowed set to 2). These trimmed reads were used in a *de novo* assembly in CLC Genomics workbench with default settings, and the resultant contigs were used for BLAST searches as necessary. *De novo* assembly of the trimmed raw reads yielded a genome consisting of 10740 contigs with a N50 = 1909 bp and average coverage of 112x.

### PCR and Sanger Sequence Analyses of the *MAT* Locus

To reconstruct the *MAT-1* locus, a multifaceted approach was utilized. PCR and Sanger sequence analyses were used to check and verify the presence and organization of specific genes and regions in the various self-fertile and self-sterile representatives of isolate CMW14799. The data were then used in conjunction with the genome sequences to assemble the full locus that included the *MAT-1* region for both self-fertile and self-sterile isolate types.

Primer pairs to amplify the predicted *MAT-1* genes and the regions flanking these were designed using the online primer design tool Primer3 [62] (Table S2). These primer pairs were used to amplify fragments of the corresponding regions in all isolates. To do this, DNA was isolated from all cultures as described before [61]. Each 25 μl reaction mixture 1 U Roche Fast-Start *Taq* mixture and reaction buffer (Roche, Mannheim, Germany), 2.5 mM MgCl_2_, 0.25 mM of each dNTP, 0.2 mM of each primer and 20–50 ng of template DNA. Amplification reactions were performed on an Eppendorf Thermocycler (Eppendorf AG, Mannheim, Germany). Following 5 min at 96°C, PCRs consisted of 35 cycles of 30 s at 95°C, 30 s at 50°C, and 1 min at 72°C, and a final extension step at 72°C for 7 min. Products were visualised using standard agarose (LE Agarose, SeaKem, Rockland, USA) gel electrophoresis [63]. Selected amplicons were purified using the DNA Clean & Concentrator kit (Zymo Research Corporation, CA, U.S.A.) and sequenced using the original PCR primers, the Big Dye cycle sequencing kit with Amplitaq DNA polymerase (Perkin-Elmer, Warrington, UK) and an ABI PRISM 3300 Genetic Analyser (Applied Biosystems, Foster City, USA).

The self-fertile isolate CMW14799 and its self-sterile counterpart, CMW14799_B11, were used in long-range PCR and sequencing reactions to determine the structure of the mating type locus. To achieve this, primer sets 2863R/MAT1-2-1R, MAT1-2-1F/Primer12 and 2863R/Primer12 (Table S2) were used to determine the arrangement of the *MAT1-1-1*, *MAT1-2-1* and *MAT1-1-2* genes within the locus. These PCRs were performed in 50 μl volumes using the Expand Long Range PCR kit (Roche, Mannheim, Germany) according to the manufacturer’s protocol, using a 50°C annealing temperature and optimizing for products in a 3–5 kb size range. These products were purified as before and cloned using the pGem-T Easy cloning kit (Promega, Madison, USA). Plasmids containing the fragment of interest were extracted using the PureYield Plasmid Miniprep System (Promega, Madison, USA) and directly subjected to sequencing as described above using the vector-specific primers T7 and SP6 [64], [65]. Where needed, additional sequencing primers were designed based on the sequences produced using Primer3.

All chromatograms were visualized and analysed using the CLC Main Workbench v6.6.2 software package (CLC bio, Denmark). This package was also used to assemble the different *MAT-1* gene sequences into contigs. These contigs were compared with each other to determine which section had been lost during switching, while repeat regions were identified using the RepFind program [37]. The deleted region including an additional 500 bases flanking it on either side was submitted to RepFind using the following input values: P-value cutoff = 0.0001, minimum repeat length = 10, maximum repeat length = infinity, low complexity filter = on, background Markov model = first order. Statistical support was evaluated using the query sequence and E-values as produced by the program. To determine whether any other repeat regions were present in the extended region of the self-fertile isolate, we used the online interface of REPuter [38]. The full KF033902 sequence was submitted with the following parameters: Match sequence against forward, reverse, complement and reverse complement; maximum computed repeats = 50; minimal repeat size = 10, use Hamming for distance calculations. An identified 260 bp repeat region was then used as query sequence in a BLASTn search of the CMW14799 *C. fimbriata* genome [31]. The genome under NCBI accession number APWK01000000 was downloaded and the BLAST option of the CLC genomics workbench was used with the following settings: BLASTn search option with the low complexity filter on, expect value = 10.0, word size = 11, match/mismatch = 2/−3, gap costs = 5 for existence and 2 for extension. The same BLAST search was done on the *de novo* assembled version of the 14799_B11 genome using the 260 bp repeat and a 3851 bp fragment that is lost from the self-fertile genome as input queries. The repeat sequence was also subjected to a BLASTn search of the full nucleotide collection in the Genbank database (using the CLC Main Workbench and the same settings as above), as well as to CENSOR [66] (using default settings) for comparison to the Repbase repeat database.

### Comparison of the *MAT-1* Locus and Gene Arrangements

To determine whether the *MAT-1* gene arrangement observed in self-fertile isolate CMW14799 and self-sterile isolate CMW14799_B11 was conserved across multiple isolates, we amplified and sequenced the *MAT-1* region from a second self-fertile isolate CMW1547, as well as six self-sterile isolates (CMW14799_B4, CMW14799_B1, CMW14799_B3, CMW1547_B1, CMW1547_B5, and CMW1547_B6). For this purpose we used the same amplification and sequencing approaches as described above. After sequencing and assembly, the contigs of these isolates were compared with each other and with those of isolates CMW14799 and CMW14799_B11. Sequence similarity between the *MAT* regions was determined using the “create pairwise comparison” command of the CLC genomics workbench.

Finally we designed two primer sets (Primer17+Primer25 and Primer17+Primer30) to discriminate between the self-sterile and self-fertile *MAT* locus. Primer17 binds in the region between the *MAT1-1-1* and *MAT1-2-1* genes (Figure 1A) that is not lost during the switching process. Primer30 binds in a region that is lost during the switching event, while Primer25 binds in the region following the deleted region. All 19 isolates used in this study were then subjected to PCRs with these primer sets using Fast-Start *Taq* and the conditions described above. After agarose gel electrophoresis, a self-sterile *MAT-1* configuration was identified by an amplicon from primer set Primer17/Primer25, while the self-fertile configuration was characterised by an amplicon produced using primer set Primer17/Primer30. For confirmation of these results, selected representative fragments were sequenced as described above.

## Supporting Information

Figure S1 **Conservation in the conserved domains of the MAT1-1-1 (A), MAT1-1-2 (B) and MAT1-2-1 (C) proteins.** An alignment showing the amount of conservation in the (A) alpha-box domain of the MAT1-1-1 protein, (B) PPF/HPG domain of the MAT1-1-2 protein and (C) the HMG box domain of the MAT1-2-1 protein. Black arrows indicate the presence of a conserved intron in both the *MAT1-1-1* and *MAT1-2-1* genes. *indicates the position of the conserved PPF domain and ∧indicates the presence of a conserved HPG domain in the MAT1-1-2 protein. Red shading indicates amino acids with ≥80% identity and yellow indicates amino acids with <80% identity. The numbers at the end of the sequences indicate the number of amino acids for each species used in the alignments. Species used and accession numbers: *Cryphonectria parasitica MAT1-1* locus AF380365 and *MAT1-2* locus AF380364. *Neurospora crassa mat A* locus M33876 and *mat a* locus M54787. *Podospora anserina mat-* locus X73830/X64194 and *mat+* locus X64195. *Cordyceps takaomontana MAT1-1* locus AB096216 and *MAT1-2* locus AB084921. *Fusarium fujikuroi MAT1-1* locus AF100925 and *MAT1-2* locus AF100926. *Pyrenopeziza brassicae MAT1-1* locus AJ006073 and *MAT1-2* locus AJ006072. *Mycosphaerella graminicola MAT1-1* locus AF440399 and *MAT1-2* locus AF440398. *Cochliobolus heterostrophus MAT1-1* locus X68399 and *MAT1-2* locus X68398. *Ceratocystis fimbriata*, this study. *Magnaporthe grisea MAT1-1* locus AB080670 and *MAT1-2* locus AB080671.
(TIF)

Table S1 **Bi-directional BLAST analysis to identify** ***MAT*** **genes from the** ***Ceratocystis fimbriata*** **CMW14799 genome sequence.**
(DOCX)

Table S2 **Primers used to amplify fragments associated with the mating locus (** ***MAT-1*** **) in** ***Ceratocystis fimbriata*** **.**
(DOCX)

Table S3 **Repeat regions identified using RepFind and REPuter.**
(DOCX)

Table S4 **Numbers and origins of the isolates of** ***Ceratocystis fimbriata*** **used in this study.**
(DOCX)
