# Supplementary figures and images for: DNA Loss at the Ceratocystis fimbriata Mating Locus Results in Self-Sterility
Source: PLoS One. 2014 Mar 20;9(3):e92180. doi: 10.1371/journal.pone.0092180 (PMC3961304; doi:10.1371/journal.pone.0092180)

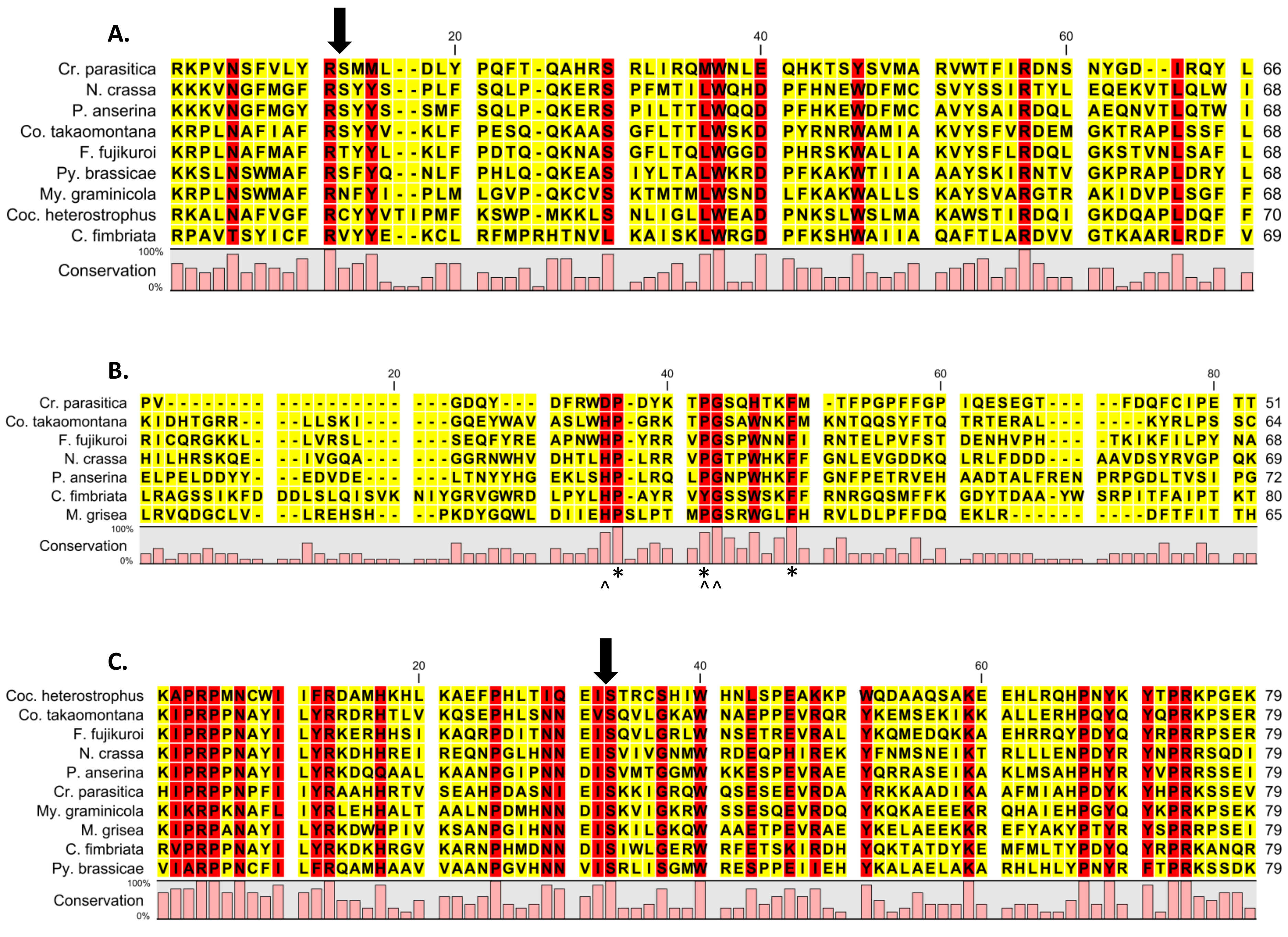

Supplement: Figure S1 — Conservation in the conserved domains of the MAT1-1-1 (A), MAT1-1-2 (B) and MAT1-2-1 (C) proteins. An alignment showing the amount of conservation in the (A) alpha-box domain of the MAT1-1-1 protein, (B) PPF/HPG domain of the MAT1-1-2 protein and (C) the HMG box domain of the MAT1-2-1 protein. Black arrows indicate the presence of a conserved intron in both the MAT1-1-1 and MAT1-2-1 genes. *indicates the position of the conserved PPF domain and ∧indicates the presence of a conserved HPG domain in the MAT1-1-2 protein. Red shading indicates amino acids with ≥80% identity and yellow indicates amino acids with <80% identity. The numbers at the end of the sequences indicate the number of amino acids for each species used in the alignments. Species used and accession numbers: Cryphonectria parasitica MAT1-1 locus AF380365 and MAT1-2 locus AF380364. Neurospora crassa mat A locus M33876 and mat a locus M54787. Podospora anserina mat- locus X73830/X64194 and mat+ locus X64195. Cordyceps takaomontana MAT1-1 locus AB096216 and MAT1-2 locus AB084921. Fusarium fujikuroi MAT1-1 locus AF100925 and MAT1-2 locus AF100926. Pyrenopeziza brassicae MAT1-1 locus AJ006073 and MAT1-2 locus AJ006072. Mycosphaerella graminicola MAT1-1 locus AF440399 and MAT1-2 locus AF440398. Cochliobolus heterostrophus MAT1-1 locus X68399 and MAT1-2 locus X68398. Ceratocystis fimbriata, this study. Magnaporthe grisea MAT1-1 locus AB080670 and MAT1-2 locus AB080671. (TIF) [file pone.0092180.s001.tif]
