# Supplementary material for: DNA Loss at the Ceratocystis fimbriata Mating Locus Results in Self-Sterility
Source: PLoS One. 2014 Mar 20;9(3):e92180. doi: 10.1371/journal.pone.0092180 (PMC3961304; doi:10.1371/journal.pone.0092180)
Supplement: Table S1 — Bi-directional BLAST analysis to identify MAT genes from the Ceratocystis fimbriata CMW14799 genome sequence. (DOCX) [file pone.0092180.s002.docx]

**Table S1: Bi-directional BLAST analysis to identify *MAT* genes from the *Ceratocystis fimbriata* CMW14799 genome sequence.**

|  | **Local tBLASTn to genome^1^** | | | | **BLASTp to NCBI database** | | |
| --- | --- | --- | --- | --- | --- | --- | --- |
|  | **Contig size^2^** | **Contig name** | **E-value** | **ORF number** | | **Best hit** | **E-value** |
| *C. fimbriata* MAT1-2-1 (AAP03161) | 3320 | Contig  02280 | 7.6x10^-27^ | 1 | | MAT-2 HMG box protein [*Ceratocystis fimbriata*] | 2x10^-28^ |
| *Fusarium graminearum* MAT1-1-2 (ABE98346) | 14339 | Contig 02573 | 7.28x10^-4^ | 1 | | MAT1-1-2 [*Trichoderma reesei*] | 3x10^-08^ |
|  |  |  |  | 2 | | Conserved hypothetical protein [*Verticillium alboatrum*] | 0.0 |
|  |  |  |  | 3 | | Importin-beta domain-containing protein [*Glomerella graminicola*] | 0.0 |
| *F. fujikuroi* MAT1-1-1 (AAC71055) | 28372 | Contig  00758^3^ | 5.08x10^-12^ | 1 | | Hypothetical protein^4^ | - |
|  |  |  |  | 2 | | Cytochrome c oxidase subunit VIa [*Aspergillus kawachii*] | 4x10^-37^ |
|  |  |  |  | 3 | | Putative DNA lyase apn2 protein [*Neurospora terricola*] | 3x10^-131^ |
|  |  |  |  | 4 | | Anaphase-promoting complex protein [*V. dahliae*] | 2x10^-150^ |
|  |  |  |  | 5 | | SLA2 [*Colletotrichum higginsianum*] | 0.0 |
|  |  |  |  | 6 | | Mating type protein 1-1-1 [*Fusarium* sp. F67] | 7x10^-13^ |
| *F. graminearum*  MAT1-1-3 (AAG42812) | 11893 | Contig 02181 | 1.19x10^-11^ | - | | N/A^4^ | N/A^5^ |

^1^ Genome used was obtained from the NCBI database under accession number APWK00000000, version CFim_1.0.

^2^ Contig sizes are indicated as the number of nucleotide bases.

^3^ This contig was present in version CFim_2.0 of the C. fimbriata genome under accession number APWK00000000.

^4^ BLASTp searched did not identify any proteins with similarity to this ORF.

^5^ Comparison of the *F. graminearum* MAT1-1-3 protein sequence to the translated nucleotide sequence of *C. fimbriata* contig 4 indicated that similarity was restricted to the HMG box region.
