# Supplementary material for: DNA Loss at the Ceratocystis fimbriata Mating Locus Results in Self-Sterility
Source: PLoS One. 2014 Mar 20;9(3):e92180. doi: 10.1371/journal.pone.0092180 (PMC3961304; doi:10.1371/journal.pone.0092180)
Supplement: Table S2 — Primers used to amplify fragments associated with the mating locus ( MAT-1 ) in Ceratocystis fimbriata . (DOCX) [file pone.0092180.s003.docx]

**Table S2: Primers used to amplify fragments associated with the mating locus (*MAT-1*) in *Ceratocystis* *fimbriata***.

| **Primer name** | **Sequence (5’-3’)** | **Nucleotide position^1^** | **PCR product size** |
| --- | --- | --- | --- |
| Cf_Mt1_F1 | GAA GTG CCT TCG CTT TAT GC | 27989…28008 | 436 bp |
| Cf_Mt1_R1 | GAC CGC GAT TCT AAC CAA AA | 27573…27592 |  |
| MAT1-1-2F | ACG CTA GGC TTA TTG CTG GA | 35120…35139 | 763 bp |
| MAT1-1-2R | GTT AAG TTG TCC CGG GGA AT | 35863…35882 |  |
| MAT1-2-1F | AAG ATG CTC TTT AAT ACC CAC CA | 31642…31664 | 495 bp |
| MAT1-2-1R | TGC CGC TAA TAA GCT AGG AA | 32117…32136 |  |
| 2863R | ATT TTC ACA TCA CAT CGC CA | 25605…25624 | 12029 bp^2^ / 8448 bp^3^ |
| Primer12 | CTG GTC TTC TGG CTG GCT AC | 37614…37633 |  |
| Primer17 | TTA GCC GGA CGC TTA TCA TT | 28632…28651 | 591 bp^2^ / 855 bp^3^ |
| Primer25 | CTC ATG ACG GAT ATT GCC AGT | 33047…33067 |  |
| Primer30 | CCC TAT CTT GGG AAA CGA GA | 29203…29222 |  |

^1^ Primer positions are based on the CMW14799 self-fertile contig accession number KF033902

^2^ Fragment size produced from a self-fertile *MAT* locus

^3^ Fragment size produced from a self-sterile *MAT* locus
