# Supplementary material for: DNA Loss at the Ceratocystis fimbriata Mating Locus Results in Self-Sterility
Source: PLoS One. 2014 Mar 20;9(3):e92180. doi: 10.1371/journal.pone.0092180 (PMC3961304; doi:10.1371/journal.pone.0092180)
Supplement: Table S3 — Repeat regions identified using RepFind and REPuter. (DOCX) [file pone.0092180.s004.docx]

**Table S3: Repeat regions identified using RepFind and REPuter**.

| **Repeat number^1^** | **Repeat length** | **Repeat copies** | **Position ^2, 3^** |
| --- | --- | --- | --- |
| 1 | 30 | 3 | 28338, 28407, 28545 |
| 2 | 26 | 4 | 28311, 28380, 28518, 28587 |
| 3 | 18 | 4 | 28350, 28419, 28488, 28557 |
| 4 | 27 | 3 | 28307, 28445, 28514 |
| 5 | 46 | 3 | 28380, 28518, 28587 |
| 6 | 260 | 2 | 28768, 32349 |
| 7 | 15 | 5 | 28284, 28353, 28422, 28491, 28560 |
| 8 | 23 | 5 | 28311, 28380, 28449, 28518, 28587 |
| 9 | 19 | 4 | 28338, 28407, 28545, 28614 |
| 10 | 25 | 3 | 28419, 28488, 28557 |
| 11 | 33 | 2 | 28304, 28511 |
| 12 | 68 | 2 | 28380, 28518 |
| 13 | 57 | 2 | 28369, 28576 |
| 14 | 48 | 3 | 33217, 33270, 33323 |

^1^ Repeats 1-13 were identified using RepFind at a P-value of 0.0. Repeat 6 and 14 were identified by REPuter at an E-value of E=1.77x10-148 and E=765x10^-21^, respectively.

^2^ Positions are based on the CMW14799 self-fertile contig accession number KF033902.

^3^ All the repeats, apart from 6 and 14 is confined to a 349 bp region of the genome located before the deleted region, from position 28284 to position 28632 of the self-fertile sequence KF033902.
