# Supplementary material for: DNA Loss at the Ceratocystis fimbriata Mating Locus Results in Self-Sterility
Source: PLoS One. 2014 Mar 20;9(3):e92180. doi: 10.1371/journal.pone.0092180 (PMC3961304; doi:10.1371/journal.pone.0092180)
Supplement: Table S4 — Numbers and origins of the isolates of Ceratocystis fimbriata used in this study. (DOCX) [file pone.0092180.s005.docx]

**Table S4: Numbers and origins of the isolates of *Ceratocystis fimbriata* used in this study.**

| **Isolate number** | **Isolate number^1^** | **Origin** | **Fertility** |
| --- | --- | --- | --- |
| 14799^2^ | CMW14799,CBS114723 | Papua New Guinea | Self-fertile |
| 14799_B1 | CMW39665 | Single spore from 14799 | Self-sterile |
| 14799_B2 | CMW39666 | " | " |
| 14799_B3 | CMW39667 | " | " |
| 14799_B4 | CMW39668 | " | " |
| 14799_B5 | CMW39960 | " | " |
| 14799_B6 | CMW39961 | " | " |
| 14799_B7 | CMW39669 | " | " |
| 14799_B8 | CMW39670 | " | " |
| 14799_B9 | CMW39671 | " | " |
| 14799_B10 | CMW39672 | " | " |
| 14799_B11 | CMW39962 | " | " |
| 1547^3^ | CMW1547,CBS123010 | USA | Self-fertile |
| 1547_B1 | CMW39673 | Single spore from 1547 | Self-sterile |
| 1547_B3 | CMW39674 | " | " |
| 1547_B4 | CMW39675 | " | " |
| 1547_B5 | CMW39676 | " | " |
| 1547_B6 | CMW39677 | " | " |
| 1547_B9 | CMW39678 | " | " |

^1^ CMW = Culture collection of the Forestry and Biotechnology Institute (FABI), University of Pretoria, South Africa; CBS = Centraalbureau voor Schimmelcultures, Utrecht, The Netherlands.

^2^ Collected by D. McNew from *Ipomoea batatas* in North Carolina, USA.

^3^ Collected by E.H.C. McKenzie and F.M. Quinn from *Ipomoea batatas* in Papua New Guinea.
